# Duodenal Brunner's gland hamartoma resected using laparoscopic and endoscopic cooperative surgery: A case report

**DOI:** 10.1016/j.ijscr.2024.110617

**Published:** 2024-11-19

**Authors:** Shota Sato, Tetsuro Kawazoe, Yasushi Tanaka, Mitsuhiko Ota, Eiji Oki, Tomoharu Yoshizumi

**Affiliations:** Department of Surgery and Science, Kyushu University, Fukuoka, Japan

**Keywords:** Brunner's gland hamartoma, D-LECS, Duodenal tumor

## Abstract

**Introduction and importance:**

Brunner's gland hamartoma is a rare benign duodenal tumor. Resection is recommended for large or symptomatic lesions, but conventional pancreaticoduodenectomy and other procedures can be overly invasive for the lesion. We report a case of Brunner's gland hamartoma resected using laparoscopic and endoscopic cooperative surgery (LECS).

**Case presentation:**

A 51-year-old woman was referred to our hospital with an asymptomatic duodenal tumor that had increased in size. A submucosal tumor was found on the anterior wall of the duodenal bulb during a detailed examination, and surgery was performed because the tumor was large (2 cm). In order to optimally resect the tumor, duodenal LECS (D-LECS) was selected. The resection line was determined while checking the base of the lesion with an intraoperative endoscope, and after the lesion was resected, the mucosal defect was closed using laparoscopic manipulation. Histopathological evaluation revealed Brunner's gland hyperplasia and mixed smooth muscle bundles, and the lesion was diagnosed as a Brunner's gland hamartoma. The surgery was completed without any problems, and the patient made a full recovery after the surgery with no complications such as stenosis, and no recurrence was observed.

**Clinical discussion:**

With D-LECS, the lesion can be resected without excess or deficiency, and the incision can be sutured with minimal invasiveness. D-LECS is an effective method as a treatment option for Brunner's gland hamartoma.

**Conclusion:**

We herein report a case of Brunner's gland hamartoma treated safely with a minimally invasive surgical technique: D-LECS.

## Highlights

• Brunner's hamartoma is a rare benign duodenal tumor.
• There are no reports of cure of Brunner's hamartoma with duodenal LECS.
• LECS for lesions close to the pyloric ring is difficult.
• A Brunner's hamartoma close to the pyloric ring could be resected with D-LECS.

## 1 Introduction

Brunner's glands are branched ductal glands located mainly in the deep mucosal or submucosal layers of the duodenal bulb and proximal duodenum [1].

In recent years, the pathogenesis of tumors in the duodenal region has been revealed [2]. Brunner's gland hamartoma is a rare benign duodenal tumor representing approximately 5–10 % of all benign tumor of the duodenum. [3,4]. Conservative treatment of small lesions is acceptable. However, in patients with large lesions and symptoms such as abdominal pain, gastrointestinal obstruction, bleeding, and anemia, endoscopic or surgical resection should be considered [4]. Endoscopic resection is recommended if the size, location, and other criteria are met. However, surgical resection is necessary when the tumors are large and their location is complicated [5]. Herein, we report a case of Brunner's gland hamartoma resected using laparoscopic and endoscopic cooperative surgery (LECS). The work has been reported in line with the SCARE criteria [6].

## 2 Case presentation

### 2.1 Patient information and clinical findings

A 51-year-old woman was referred to our hospital with an asymptomatic duodenal tumor that was noted during health checkup and displayed a tendency to increase in size compared with its size 5 years earlier. She had a history of Cesarean section without any other remarkable medical history or comorbidities; taking no medications.

### 2.2 Laboratory tests and imaging

**Fig. 1:**
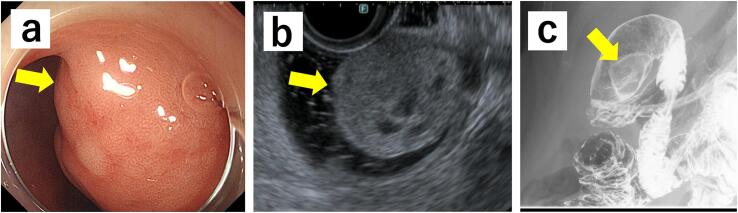
**a** Esophagogastroduodenoscopy revealed a large, 20 mm SMT-like mass on the anterior wall of the duodenal bulb (arrow). **b** It is observed as a hypoechoic, full mass measuring 30 mm × 20 mm on EUS (arrow). **c** Gastrofluoroscopy reveals a 25–30 mm, borderline, well-defined elevated lesion on the anterior wall of the bulb, without obvious wall deformity or sclerosis (arrow). EUS, endoscopic ultrasound; SMT, submucosal tumor,

Laboratory examinations revealed no abnormal findings on routine hematological and biochemical tests; tumor marker levels were within the reference ranges (carcinoembryonic antigen, 0.6 ng/ml; cancer antigen 19–9, 6.6 U/ml). Esophagogastroduodenoscopy revealed a submucosal tumor, approximately 20 mm in size, located on the anterior wall of the duodenal bulb (Fig. 1a). Endoscopic ultrasonography revealed a 30 × 20 mm hypoechoic lesion (Fig. 1b). Endoscopic ultrasound-fine needle aspiration revealed no malignant findings; leading to no histological diagnosis. In the upper gastrointestinal barium contrast examination, a protruding lesion was observed on the anterior wall of the duodenal bulb. There was no lateral deformity of the duodenal wall, and the tumor was located 1 cm distal to the pyloric ring, and was distant from the duodenal papilla (Fig. 1c). Contrast-enhanced computed tomography (CT) revealed a 2-cm elevated lesion in the duodenal bulb without invasion into the surrounding tissue.

### 2.3 Treatment and surgical intervention

Duodenal submucosal tumor was diagnosed based on these findings. The tumor was 2 cm in diameter; the possibility of a carcinoma in adenoma could not be ruled out. Therefore, surgery was performed. The tumor was too large to be resected endoscopically. However, determining the extent of resection in laparoscopic surgery was difficult; there was a concern of excessive resection. Therefore, duodenal LECS (D-LECS) was selected to ensure that the tumor could be optimally resected.

**Fig. 2:**
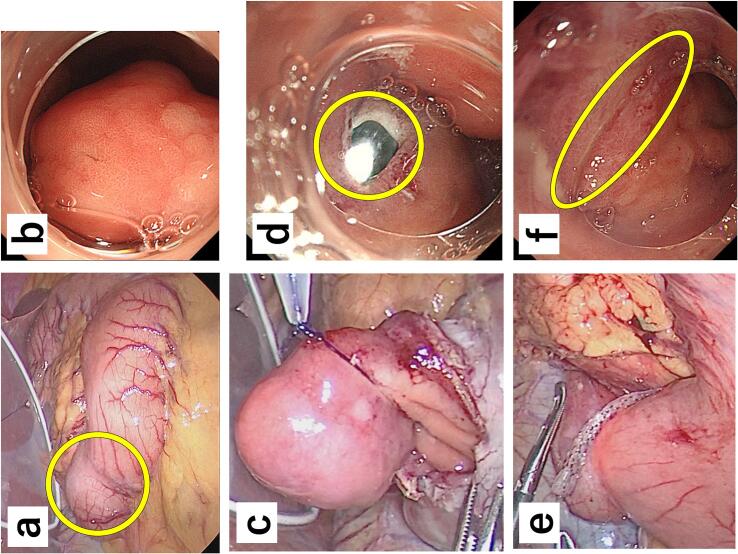
**a** Intraoperative findings reveals a mass lateral to the mouth of the pyloric ring (circle). **b** Esophagogastroduodenoscopy reveals a mass at the mouth of the pyloric ring. **c** The lesion is resected with traction using an endoloop. **d** All layers of the lesion are incised from the mucosal surface to the serosa (circle). **e** The mucosal defect is sutured. **f** Intraoperative esophagogastroduodenoscopy confirms no bleeding or leakage (circle).

Both laparoscopic and endoscopic examinations revealed a tumor just beside the pyloric ring on the duodenal side. (Fig. 2a). The base was located on the anterior wall of the duodenal bulb. (Fig. 2b). An incision was made from the mucosal surface to the serosa after determining the line of dissection along the base of the tumor, with intraoperative endoscopic confirmation. The incision was widened via laparoscopic manipulation using laparoscopic coagulating shears; resection was performed along the contour of the tumor while suspending the tumor with ENDOLOOP® ligature as appropriate (Fig. 2c). The defect was temporarily closed along the short axis and stapled using a linear stapling device (Fig. 2d,e). The surgery was completed after confirming that there was no bleeding or leakage on laparoscopy or endoscopy (Fig. 2f), respectively, and that there was no obstruction on intraoperative endoscopy. The operative time was 152 min; the volume of blood loss was minimal.

### 2.4 Follow-up and outcome

**Fig. 3:**
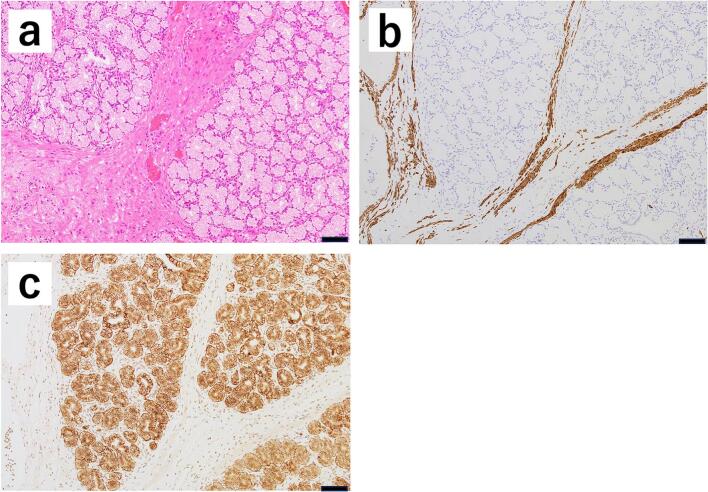
**a** Histopathological evaluation shows a proliferation of Brunner's glands (scale bar: 100 μm). **b** Desmin staining shows abnormal proliferation of Brunner's glands mixed with smooth muscle bundles (scale bar: 100 μm). **c** MUC6 staining shows abnormal proliferation of Brunner's glands (scale bar: 100 μm).

Histopathological evaluation showed the proliferation of Brunner's glands stained with MUC6; desmin staining showed mixed smooth muscle bundles, leading to the diagnosis of Brunner's gland hamartoma (Fig. 3). After the surgery, she recovered without any complications, and was able to eat without any problems. An upper gastrointestinal endoscopy was performed one week after the surgery, and there were no signs of stenosis or suture failure. She was discharged from the hospital two weeks after the surgery. She was regularly monitored as an outpatient, and at one year after the surgery, there were no signs of stenosis or recurrence.

## 3 Discussion

Brunner's gland hamartoma and Brunner's gland hyperplasia are rare diseases [3].

Microscopically, Brunner's gland hyperplasia is characterized by a neutral mucin-containing gland, occupying at least 50.0 % of the duodenal mucosa [1]. In contrast, Brunner's gland hamartoma refers to proliferating glands with cystic dilatation and smooth muscle mixed with adipose tissue, large ducts, and infiltrating lymphocytes [1]. The key difference between the two is that the glands are mixed with other benign components [7]. Immunohistochemically, Brunner's glands show elevated levels of MUC6; some dilated or keratinized Brunner's glands simultaneously express MUC5AC [8].

Brunner's gland hamartomas or hyperplasias are usually benign [3]. However, in a previous report, dysplasia was found in 2.1 % of 722 Brunner's gland hyperplastic lesions and invasive carcinoma in 0.3 % [9]. Conservative treatment of small lesions is acceptable, whereas endoscopic or surgical resection is recommended for large or symptomatic lesions [10]. Endoscopic submucosal dissection (ESD) can be used to completely resect the lesion. However, the risk of intraoperative and delayed perforation is higher than that with endoscopic mucosal resection [11]. Large ulceration caused by ESD and chemical irritation from pancreatic juice and bile may lead to delayed perforation [11]. The endoscopic resection of large lesions is often difficult. The duodenal cavity is particularly narrow, resulting in poor visibility and maneuverability. In addition, the mass may be transported to the distal end by intestinal peristalsis [12]. In contrast, conventional surgical procedures, such as pancreatoduodenectomy or segmental duodenectomy, may be excessively invasive for early duodenal tumors and are associated with high rates of serious postoperative complications and decline in patient's quality of life [13,14]. Laparoscopic local resection is a minimally invasive treatment of early duodenal tumors [15].It is very effective in managing lesions protruding on the serosal side. However, regarding intraluminal tumors, determining an appropriate resection line from the serosal surface laparoscopically is difficult. Inadequate resection margins may cause local recurrence, whereas massive resections with excessive margins may result in deformities or stenosis [15].

In contrast to the abovementioned procedures, D-LECS allows for local whole-layer resection without excess or deficiency of the excisional area; the excisional wound can be sutured in a minimally invasive manner [16]. LECS in the duodenum has been reported to be useful for lesions that do not involve the papillary region and is covered by insurance in Japan since 2020 [17]. In this case, the lesion was captured from the serous membrane side using endoscopic manipulation, and it was possible to perform an appropriate resection with a resection margin along the outline of the lesion.

LECS for Brunner's gland hyperplasia was reported in one case, and the operation was completed without complications and the postoperative course was good [18]. To the best of our knowledge, there have been no reports of LECS for Brunner's gland hamartoma. D-LECS allows for caudal transfer of the transverse colon and the Kocher maneuver of the duodenum, which enables adequate manipulation of the duodenum and ensures confirmation and reinforcement of the thin duodenal wall after endoscopic resection [18]. In this case, Kocher maneuver and caudal displacement of the transverse colon were not necessary, but it was necessary to incise the hepatoduodenal ligament to improve the mobility of the duodenum when resecting the lesion. In addition, the lesion and pyloric ring were close together in this case, with a distance of only 1 cm. Lesions close to the pyloric ring increase the difficulty of endoscopic manipulation and may cause postoperative deformity, stenosis and impaired transit due to reduced pyloric ring function. In our case, although distal gastrectomy (DG) was considered instead of LECS, LECS was chosen because the lesion did not extend beyond the pyloric ring and it, itself, was relatively small (2 cm). If the lesion extends into the pyloric ring or if a large mucosal defect is expected due to the size of the lesion or the shape of its base, other surgical procedures such as pyloric gastrectomy, pancreaticoduodenectomy, or duodenal segmental resection should be selected.

One issue in D-LECS is the exposure of intestinal fluid and tumor to the abdominal cavity due to the incision of the duodenum. Regarding determining the optimal line of dissection, opening the duodenum and identifying the tumor base are important. In this case, the ENDOLOOP® ligature was suspended over the base of the lesion, allowing the tumor to be pulled and manipulated in a patulous manner, preventing intra-abdominal contamination and improving intraoperative maneuverability. The ENDOLOOP® ligature also made clearly visualizing the tumor base possible and was a useful technique in terms of minimal mucosal loss. Since the use of D-LECS becomes more prevalent, further refining the technique and intraoperative positions will be necessary.

## 4 Conclusions

Here, we report a case of Brunner's hamartoma treated using a minimally invasive surgical technique called D-LECS. To perform D-LECS more safely and optimize the technique, accumulating more cases in the future is necessary.

## Abbreviations

CT: computed tomography
LECS: laparoscopic and endoscopic cooperative surgery
D-LECS: duodenal laparoscopic and endoscopic cooperative surgery
ESD: Endoscopic submucosal dissection

## Registration of research studies

This case report is not registered as a ‘First in Man’ study at this time.

## CRediT authorship contribution statement

SS and TK reported the case and wrote the manuscript. MO, TK and SS were engaged in the patient's care including the surgery. YT and TY helped in drafting the manuscript. EO participated in revising the manuscript critically. All authors have read and approved the final manuscript for publication.

## Consent for publication

Written informed consent was obtained from the patient for publication of this case report and any accompanying images.

## Guarantor

Tetsuro Kawazoe, MD, PhD.

## Ethics approval and consent to participate

Not applicable.

## Funding

This research did not receive any specific grant from funding agencies in the public, commercial, or not-for-profit sectors.

## Declaration of competing interest

The authors declare that they have no competing interests.

## Data Availability

We declare that all data contained in this paper are available.
